# DNA Sequencing Diagnosis of Off-Season Spirochetemia with Low Bacterial Density in *Borrelia burgdorferi* and *Borrelia miyamotoi* Infections

**DOI:** 10.3390/ijms150711364

**Published:** 2014-06-25

**Authors:** Sin Hang Lee, Jessica S. Vigliotti, Veronica S. Vigliotti, William Jones, Thomas A. Moorcroft, Katherine Lantsman

**Affiliations:** 1 Department of Pathology, Milford Hospital, 300 Seaside Ave., Milford, CT 06460, USA; E-Mails: jessvigs@yahoo.com (J.S.V.); ronnie313vig@yahoo.com (V.S.V.); will.jones@milfordhospital.org (W.J.); 2 Origins of Health, LLC, 279 New Britain Road Berlin, CT 06037, USA; E-Mail: dr_tom@originsofhealth.com; 3 My Path Medical, 20 Park Plaza, 804, Boston, MA 02116, USA; E-Mail: klantsman@mypathmedical.com

**Keywords:** DNA sequencing, same-nested PCR, *Borrelia burgdorferi*, *Borrelia miyamotoi*, 16S rDNA, off-season spirochetemia, Lyme disease

## Abstract

A highly conserved 357-bp segment of the 16S ribosomal RNA gene (16S rDNA) of *Borrelia burgdorferi* sensu lato and the correspondent 358-bp segment of the *Borrelia miyamotoi* gene were amplified by a single pair of nested polymerase chain reaction (PCR) primers for detection, and the amplicons were used as the templates for direct Sanger DNA sequencing. Reliable molecular diagnosis of these borreliae was confirmed by sequence alignment analysis of the hypervariable regions of the PCR amplicon, using the Basic Local Alignment Search Tool (BLAST) provided by the GenBank. This methodology can detect and confirm *B. burgdorferi* and *B. miyamotoi* in blood samples of patients with off-season spirochetemia of low bacterial density. We found four *B. miyamotoi* infections among 14 patients with spirochetemia, including one patient co-infected by both *B. miyamotoi* and *B. burgdorferi* in a winter month when human exposure to tick bites is very limited in the Northeast of the U.S.A. We conclude that sensitive and reliable tests for these two Borrelia species should be implemented in the microbiology laboratory of hospitals located in the disease-endemic areas, for timely diagnosis and appropriate treatment of the patients at an early stage of the infection to prevent potential tissue damages.

## 1. Introduction

Reliable diagnosis of Lyme disease caused by *Borrelia* *burgdorferi* at its early stage of infection is important for the timely implementation of appropriate treatments to prevent tissue damages and to achieve a “cure” of the disease [1]. However, the diagnosis of Lyme disease is primarily based on evaluating the highly variable symptoms, physical findings (e.g., rash), and the possibility of exposure to infected ticks [2,3]. The commonly used 2-tier serology laboratory test which usually only turns positive during convalescence of the infection is reported to be negative or non-diagnostic in 75% of the “clinically confirmed” cases of early Lyme disease [4]. Blood culture at the stage of bacteremia has met only limited success in specialized laboratories and offers little help to the timely management of patients suffering from early Lyme disease bacterial infection due to the slow growth rate of the spirochetes in artificial media [5]. Conventional polymerase chain reaction (PCR) amplification of the bacterial DNA for detection is not sensitive enough for routine diagnostic purpose because the copies of the target DNA extracted from the very low number of spirochetes in the patient blood samples are often below the limit of detection [6,7]. False positive PCR results have also been reported, and may be due to non-specific PCR amplification of irrelevant DNA in the patient samples [8,9]. Using species-specific primers, such as the LD1 and LD2 [10,11], and TEC1 [12] primers to amplify a highly conserved segment of the 16S ribosomal RNA gene DNA (16S rDNA) of *B. burgdorferi* sensu lato for detection, followed by direct DNA sequencing of the nested PCR amplicon for validation, has been used to provide sensitive and specific molecular diagnosis of Lyme arthritis [13] and early Lyme disease at the spirochetemic stage [14].

Recently, human infections by *Borrelia miyamotoi*—a spirochete distantly related to *B. burgdorferi*, but classified in the relapsing fever Borrelia group—have been reported in the United States [15,16]. Since *B. miyamotoi* is transmitted by the same *Ixodes* vector as *B. burgdorferi* sensu stricto [17], and may cause clinical symptoms similar to those of Lyme disease, including skin rashes [18], *B. miyamotoi* infection should be included in the differential diagnosis of patients presenting with unexplained fever, headache and myalgia with or without skin rash in Lyme disease-endemic areas during the seasons when ticks are active. Molecular assays are the only option to test for *B. miyamotoi* infection because this spirochete is hard to culture in modified Barbour-Stoenner-Kelly medium and the currently available two-tier serology test offers little help for its diagnosis [15,18].

In this paper, we introduce a pair of common PCR primers for same-nested PCR amplification of a highly conserved segment with hypervariable regions of the 16S rDNA of *B. burgdorferi* and *B. miyamotoi* followed by direct DNA sequencing of the PCR amplicon for the molecular diagnosis of these two borrelial infections in patients with spirochetemia. This highly sensitive, DNA sequencing-based test can even diagnose off-season spirochetemias with low bacterial density in the deep winter months in the Northeast of the United States when human exposure to tick bites was very limited.

## 2. Results and Discussion

Nested PCR amplification of a 600-nucleotide fragment of the 16S rDNA followed by direct DNA sequencing of the PCR amplicon has been used for construction of the phylogenetic tree of various Borrelia species [18,19]. One obstacle in transferring this methodology to diagnosing Lyme disease for patient care is the very low number of spirochetes in the blood of the patients even at the spirochetemic stage of the infection [6,20], and *B. burgdorferi* sensu stricto contains only one copy of 16S rDNA per cell [21]. PCR amplification of a 600-bp target DNA for direct DNA sequencing is technically challenging in a routine diagnostic laboratory because the sensitivity of 16S rDNA PCR amplification in diagnostic clinical microbiology is inversely related to the size of the PCR amplicon [22]. In addition, to construct the phylogenetic tree it is desirable to perform DNA sequencing on a segment of the gene with highly variable regions for its discriminatory power to distinguish different closely related species. The 600-nucleotide fragment of 16S rDNA selected for borrelial speciation [18,19] encompasses the highly discriminatory V-regions 1, 2, 3 and 4 of the 16S rDNA [23]. However, for molecular diagnostic nucleic acid amplification tests it is more beneficial to target less species-specific regions for amplification so that one fixed set of primers may detect as many clinically relevant pathogens of the same group as possible for timely diagnosis and patient management. For the latter reason, clinical laboratory diagnosticians are most interested in developing general PCR primers capable of amplifying a highly conserved segment of the 16S rDNA for all of the three tick-borne pathogenic borrelial species known to be prevalent in Europe and in the U.S.A., namely the species of *B. burgdorferi* sensu stricto, *B. garinii* and *B. afzelii*, often collectively referred to as *B. burgdorferi* sensu lato [24].

As previously reported, the species-specific LD1/LD2 primer pair [10,11] and the TEC1/LD2 [12] primer pair generated a 351-bp primary PCR amplicon and a 293-bp heminested PCR amplicon, respectively, when 16S rDNA of *B. burgdorferi* sensu lato was present in the primary PCR mixture as the template [13,14]. The LD1/LD2 primer-defined segment includes the V-regions 5 and 6 which are considered to be not very discriminatory and not depended on for phylogenetic tree construction [23]. For the diagnosis of infectious agents of Lyme disease these primers are quite useful, and the signature sequence of the *B. burgdorferi* 16S rDNA can be validated by direct DNA sequencing of the nested PCR amplicon [13,14]. However, these species-specific PCR primers designed for amplification of the 16S rDNA of *B. burgdorferi* sensu lato cannot be used to amplify a correspondent DNA segment of the *B. miyamotoi* 16S rDNA. Our first task was to find a pair of general PCR primers suitable for amplification of a highly conserved segment with hypervariable regions of the 16S rDNA of both *B. burgdorferi* sensu lato and *B. miyamotoi* species around the V-regions 5 and 6 for clinical diagnostic application.

### 2.1. The M1 and M2 General PCR Primers

In the course of searching for a set of PCR primers to amplify a signature sequence of *B. miyamotoi*, the pair of M1 (5'-ACGATGCACACTTGGTGTTAA-3') and M2 (5'-TCCGACTTATCACCGGCAGTC-3') primers was found to flank a highly conserved 315-bp segment with hypervariable regions of the 16S rDNA of the various species of the *B. burgdorferi* sensu lato complex and a correspondent 316-bp segment of the 16S rDNA of *B. miyamotoi*. The hypervariable inter-primer regions of the 16S rDNA of the various species of the *B. burgdorferi* sensu lato complex and a correspondent segment of the borreliae in the relapsing fever group are summarized in Table 1.

As shown in Table 1, alignment of the sequences retrieved from the GenBank, using *B. miyamotoi* (Locus ID #JF951379.1) as the position reference, a base gap at position 770 and the unique bases at positions 764, 765, 923, 943, 967, 1043, 1062 and 1078 within this highly conserved sequence distinguish the *B. burgdorferi* sensu lato complex from those borreliae in the relapsing fever group, including *B. miyamotoi* which has its three exclusive invariant bases, namely a T at position 817, an A at position 826 and a C at position 999, respectively.

**Figure 1:**
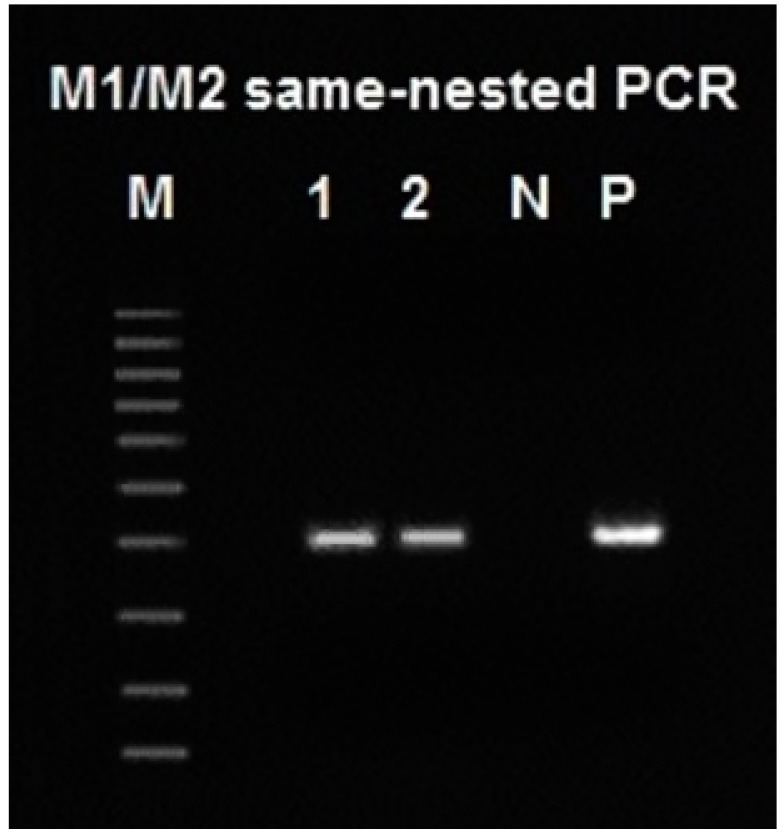
Agarose gel electrophoresis showing the M1/M2 primer-defined same-nested PCR amplicon. M = molecular ruler, 100–1000 bp; Lanes 1 and 2 = *B. miyamotoi* 16S rDNA M1/M2-defined PCR amplicon, in duplicate; N = negative water control; P = ATCC 53210 *B. burgdorferi* positive control.

We used archived ticks infected with *B. miyamotoi* and a *B. burgdorferi* pure culture as the sources of the borrelial genomic DNA for method development and found that the M1/M2 primer pair consistently amplified a 358-bp segment of the 16S rDNA extracted from *B. miyamotoi* in *Ixodes scapularis* ticks removed from the skin bites of patients and a 357-bp segment of the 16S rDNA extracted from pure culture of *B. burgdorferi* (Figure 1).

### 2.2. Direct DNA Sequencing of the M1 and M2 PCR Amplicons of B. miyamotoi and B. burgdorferi

**Figure 2:**
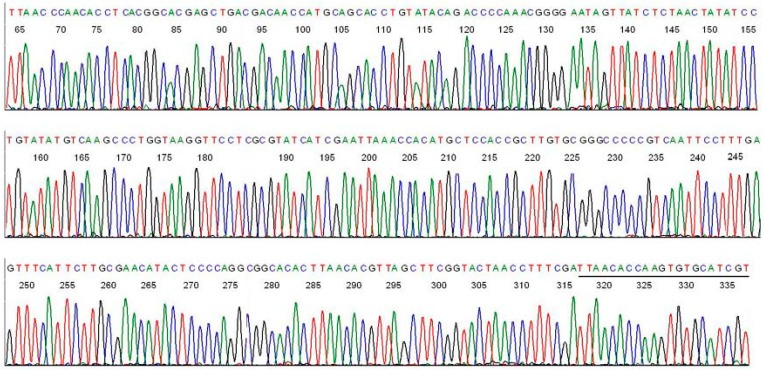
Base-calling sequencing electropherogram of an M1/M2 PCR amplicon showing part of a 358-bp segment of the 16S rDNA of *B. miyamotoi* in an *Ixodes scapularis* tick removed from the skin bite of a patient (Figure 1, lane 1). M2 was the sequencing primer. The M1 binding site is underlined.

Direct DNA sequencing of the *B. miyamotoi* PCR amplicon, using the M2 primer as the reverse sequencing primer, showed the unique hypervariable regions of the *B. miyamotoi* 16S rDNA immediately downstream of the M1 primer binding site (Figure 2).

**Table 1:** Alignment of the hypervariable bases in a highly conserved borrelial 16S rDNA sequence.

Borrelia Locus ID#	Variable Nucleotide Bases Using Borrelia miyamotoi JF951379.1 as Position Reference																						
**RF Group**	**764–766**	**768**	**770**	**817**	**826**	**856**	**898**	**900**	**908**	**923**	**929**	**943**	**946**	**949**	**960**	**963**	**999**	**1040**	**1043**	**1062**	**1064**	**1077**	**1078**
miyamotoi JF951379.1	TCG	A	G	T	A	C	G	A	A	C	A	C	T	C	T	G	C	G	G	A	A	C	G
hermsii DQ855530.1	TCG	A	G	C	G	C	G	A	A	C	G	C	T	C	T	G	T	A	G	A	A	C	G
coriaceae AF210136.1	TCG	A	G	C	G	C	A	A	A	C	G	C	T	C	T	G	T	A	G	A	A	T	G
theileri DQ872186.1	TCG	A	G	C	G	C	G	A	A	C	A	C	C	C	T	G	T	G	G	A	A	C	G
duttonii AB113315.1	TCG	A	G	C	G	C	G	A	A	C	G	C	T	C	T	G	T	G	G	A	A	C	G
parkeri AY604975.1 ^a^	TCG	A	G	C	G	C	G	A	A	C	G	C	T	C	T	G	T	A	G	A	A	C	G
turicatae U42299.1 ^a^	TCG	A	G	C	G	C	G	A	A	C	G	C	T	C	T	G	T	A	G	A	A	C	G
crocidurae AY604977.1 ^a^	TCG	A	G	C	G	C	G	A	A	C	G	C	T	C	T	G	T	A	G	A	A	C	G
lonestari AY442141.1	TCG	A	G	C	G	C	G	A	A	C	A	C	C	T	T	G	T	G	G	A	A	C	G
recurrentis CP000993.1	TCG	G	G	C	G	T	G	A	A	C	G	C	T	C	T	G	T	A	G	A	A	C	G
**BB sensu lato Complex**																							
burgdorferi NR_03929.1 ^b^	CTA	A	-	C	G	C	G	A	A	T	A	T	T	C	T	A	T	G	A	T	G	T	A
valaisiana AB091815.1	CTG	A	-	C	G	C	G	A	A	T	A	T	T	C	T	A	T	G	A	T	A	T	A
spielmanii HE582779.1	CTA	A	-	C	G	C	G	A	A	T	A	T	T	C	C	A	T	G	A	T	A	T	A
afzelii NR_074840.1	CTA	A	-	C	G	C	G	A	A	T	A	T	T	C	T	A	T	G	A	T	A	T	A
lusitaniae AB091822.1	CTA	C	-	C	G	C	G	A	A	T	A	T	T	C	T	A	T	G	A	T	G	T	A

RF = Relapsing Fever; BB = *Borrelia burgdorferi*. “-” = gap. The nucleotide sequences used for base alignment in this table were retrieved from the National Center for Biotechnology Information database; The nucleotide bases T, A and C at positions 817, 826 and 999, respectively, are unique for *B. miyamotoi*. ^a^ Discordant 16S rDNA is the accepted basis for borrelial species differentiation. However, isolates of different relapsing fever species with an identical DNA sequence in this highly conserved 16S rDNA segment can be found in the Genbank database, as exemplified here; ^b^ The species of *B. burgdorferi* sensu stricto, *B. bissettii* (NR_102956.1), *B. garinii* (NR_074854.1), and * B. americana* (HM802226.1) have an identical DNA sequence in this highly conserved 16S rDNA segment.

**Figure 3:**
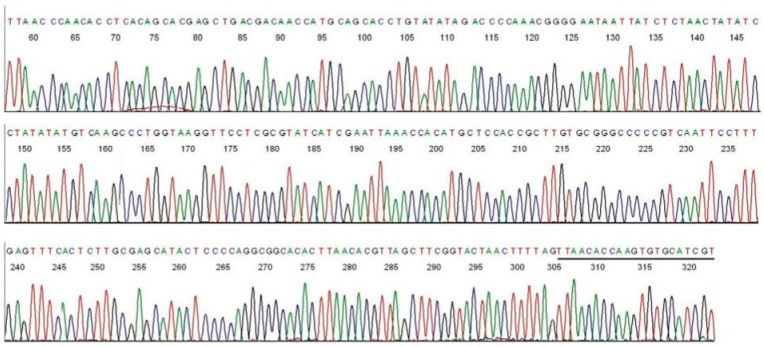
Base-calling sequencing electropherogram of an M1/M2 PCR amplicon showing part of a 357-bp segment of the 16S rDNA of *B. burgdorferi* (Figure 1, lane P). M2 was the sequencing primer. The M1 binding site is underlined.

In comparison, a direct DNA sequencing of the *B. burgdorferi* M1/M2 PCR amplicon, using the M2 primer as the reverse sequencing primer, showed the unique hypervariable regions of the *B. burgdorferi* 16S rDNA immediately downstream of the M1 primer binding site (Figure 3).

**Figure 4:**
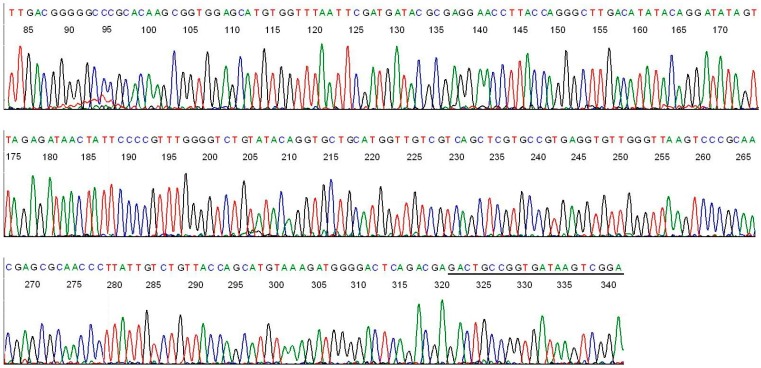
Base-calling sequencing electropherogram of an M1/M2 PCR amplicon showing part of a 358-bp segment of the 16S rDNA of *B. miyamotoi* in an *Ixodes scapularis* tick removed from the skin bite of a patient (Figure 1, lane 1). M1 was the sequencing primer. The M2 binding site is underlined (the “T” in the 5th position from the right is a mismatched base of the primer sequence).

**Figure 5:**
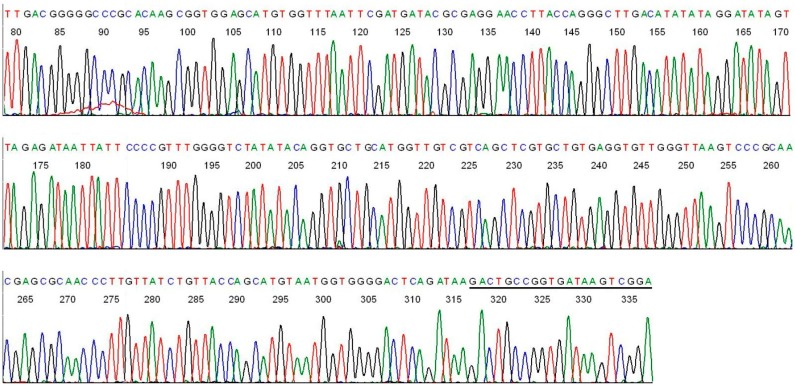
Base-calling sequencing electropherogram of an M1/M2 same-nested PCR amplicon showing part of a 357-bp segment of the 16S rDNA of *B. burgdorferi* (Figure 1, lane P). M1 was the sequencing primer. The M2 binding site is underlined.

In order to confirm the entire sequence of the M1/M2 defined amplicon of *B. miyamotoi* and that of *B. burgdorferi*, direct DNA sequencing with the M1 forward sequencing primer from the opposite direction was also performed on the nested PCR products illustrated in Figure 1. The results of the forward primer sequencing are presented in Figure 4 and Figure 5.

Connecting the two sequences illustrated in Figure 2 and Figure 4 after all of the complementary bases were converted to those for a 5'-3' reading resulted in a composite segment of 358-base sequence as follows.

ACGATGCACACTTGGTGTTAATCGAAAGGTTAGTACCGAAGCTAACGTGTTAAGTGTGCCGCCTGGGGAGTATGTTCGCAAGAATGAAACTCAAAGGAATTGACGGGGGCCCGCACAAGCGGTGGAGCATGTGGTTTAATTCGATGATACGCGAGGAACCTTACCAGGGCTTGACATATACAGGATATAGTTAGAGATAACTATTCCCCGTTTGGGGTCTGTATACAGGTGCTGCATGGTTGTCGTCAGCTCGTGCCGTGAGGTGTTGGGTTAAGTCCCGCAACGAGCGCAACCCTTGTTGTCTGTTACCAGCATGTAAAGATGGGGACTCAGACGAGACTGCCGGTGATAAGCCGGA

**Figure 6:**
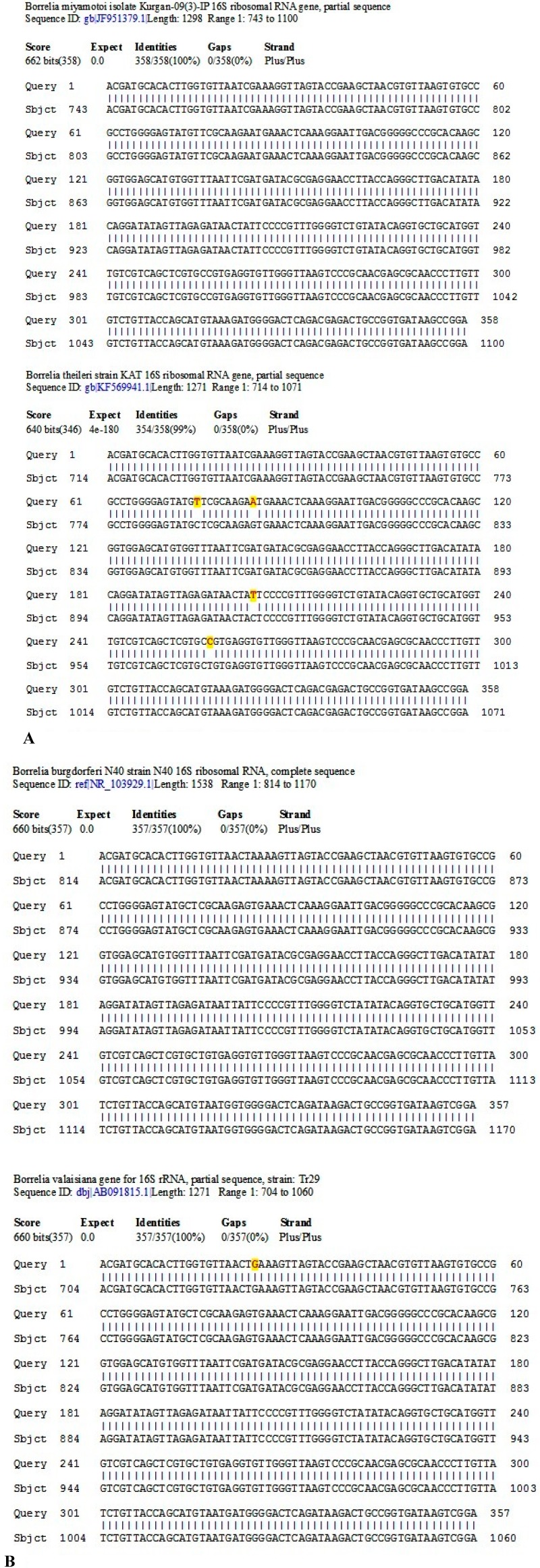
(**A**) A BLAST sequence alignment report from the GenBank validates the sequences illustrated in Figure 2 and Figure 4 are unique partial sequences of the 16S ribosomal RNA gene of the species of *B. miyamotoi* with a 100% ID match (**upper half**); and the next closest match with 4 discordant bases (high-lighted with red and yellow colors) is that of the 16S ribosomal RNA gene of a *B. theileri* strain (**lower half**); (**B**) A BLAST sequence alignment report from the GenBank validates the sequences illustrated in Figure 3 and Figure 5 are unique partial sequences of the 16S ribosomal RNA gene of the *B. burgdorferi* sensu lato complex with a 100% ID match (**upper half**); and the next closest match with one discordant base (high-lighted with red and yellow colors) is that of the 16S ribosomal RNA gene of a *B. valaisiana* strain (**lower half**).

Submission of this 358-base sequence to the GenBank for BLAST alignment analysis generated a returned report, confirming that this sequence is a unique 100% ID match with a sequence of *B. miyamotoi* 16S ribosomal RNA gene (Figure 6A).

Connecting the two sequences illustrated in Figure 3 and Figure 5 after all of the complementary bases were converted to those for a 5'-3' reading resulted in a composite segment of 357-base sequence as follows.

ACGATGCACACTTGGTGTTAACTAAAAGTTAGTACCGAAGCTAACGTGTTAAGTGTGCCGCCTGGGGAGTATGCTCGCAAGAGTGAAACTCAAAGGAATTGACGGGGGCCCGCACAAGCGGTGGAGCATGTGGTTTAATTCGATGATACGCGAGGAACCTTACCAGGGCTTGACATATATAGGATATAGTTAGAGATAATTATTCCCCGTTTGGGGTCTATATACAGGTGCTGCATGGTTGTCGTCAGCTCGTGCTGTGAGGTGTTGGGTTAAGTCCCGCAACGAGCGCAACCCTTGTTATCTGTTACCAGCATGTAATGGTGGGGACTCAGATAAGACTGCCGGTGATAAGTCGGA

Submission of this 357-base sequence to the GenBank for BLAST alignment analysis generated a returned report, confirming that this sequence is a unique 100% ID match with a sequence of *B. burgdorferi* sensu lato 16S ribosomal RNA gene (Figure 6B).

An unambiguous DNA sequence of 100–110 bases immediately downstream of the M1 primer (Table 1) excised from a base-calling Sanger sequencing electropherogram of the M1/M2 PCR amplicon with a unique 100% ID match with the standard sequence catalogued in the GenBank provides a molecular diagnosis of a strain of *B. burgdorferi* sensu lato or a strain of *B. miyamotoi* beyond reasonable doubt.

### 2.3. M1/M2 Same-Nested PCR Detection of B. miyamotoi and B. burgdorferi in Blood Samples

**Figure 7:**
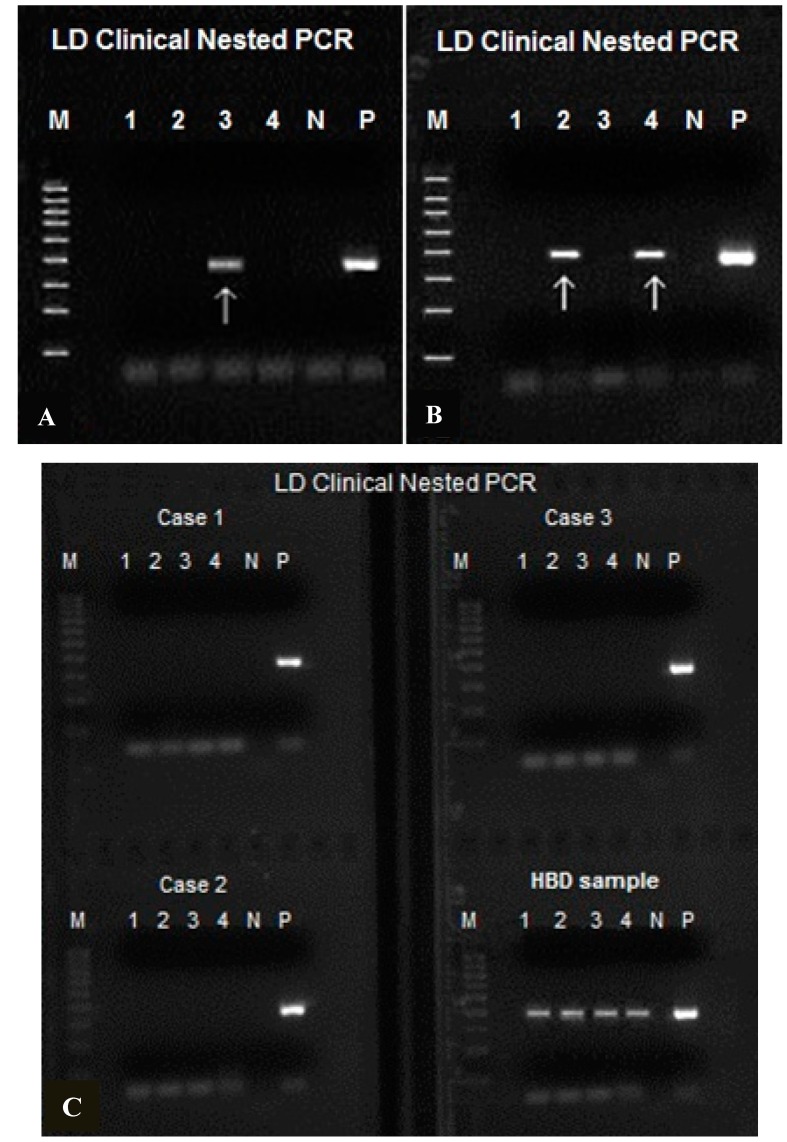
Agarose gel electrophoresis showing spirochetemias with low and high bacterial density. The DNA of the spirochetes pelleted from 1 mL of platelet-rich plasma of the patients was extracted and reduced to 100 µL in volume. One µL of the latter crude DNA concentrate was used to initiate each M1/M2 same-nested PCR ×4. (**A**) Only 1 (pointed by arrow) of 4 nested PCRs showed a target DNA amplicon, indicating that the DNA concentrate derived from 1 mL of plasma contained ~25 copies of amplifiable borrelial 16S rDNA; (**B**) Two of 4 nested PCRs generated amplicon bands indicating that the plasma of this patient might contain as many as 50 bacteria per mL; (**C**) One spirochetemic blood sample with high bacterial density (~2000 spirochetes per mL in this sample) showing an amplicon band in all 4 nested PCRs (HBD), compared with 3 negative samples (Cases 1–3). All nested PCR amplicons were validated by DNA sequencing to be those of *B. burgdorferi* or *B. miyamotoi*. M = molecular ruler; N = negative water control; P = *B. burgdorferi* control.

Using the M1/M2 same-nested PCR amplification followed by direct DNA sequencing technology described above, residual borrelial bacteria, including *B. burgdorferi*, *B. miyamotoi* and a novel Borrelia of uncertain clinical significance, were detected in archived serum samples from patients with suspect Lyme disease [25]. We also found human genomic DNA in the whole blood can be a powerful primer-binding PCR inhibitor when the target DNA is low in copy numbers, as suggested by other investigators [26,27]. Therefore, we developed a protocol to first concentrate the spirochetes in the platelet-rich plasma separated from whole blood samples by differential centrifugation before DNA extraction to increase the sensitivity of detection (see Section 3.2. Blood sample preparation). Performing nested PCR on the DNA extract from the pellet of the platelet-rich plasma may diagnose spirochetemias with a bacterial density as low as 25 per mL of whole blood (Figure 7A–C).

### 2.4. B. miyamotoi and B. burgdorferi Spirochetemias with Low Bacterial Density

Nested PCR has been found to be a necessary procedure to increase the PCR sensitivity in molecular detection of the “Lyme disease” causative agents by other investigators [28,29,30,31]. Previously, we depended on using platelet-rich plasma separated from the red cells by gravitation sedimentation as the starting material for the molecular diagnosis of spirochetemia at the early stage of *B. burgdorferi* infections [14]. The limit of detection using a one-set nested PCR on plasma samples without concentration is ~1000 spirochetes per mL of whole blood if 16S rDNA is the amplification target. By extracting the chromosomal DNA from spirochetes pelleted from 1 mL of platelet-rich plasma into 100 µL to perform four nested PCR sets per sample, the sensitivity of detection can increase markedly and the limit of detection may be as low as 25 spirochetes per mL of blood (Figure 7A). Combining the above described 4-set same-nested PCRs on concentrated platelet-rich plasma samples and direct DNA sequencing technology, a subset of patients with spirochetemia, all with a low bacterial density of 25–50 per mL of blood, have been identified in the northeastern states of the U.S.A. in the deep winter month of February 2014 when human exposure to tick bites was minimal. The key clinical information of 14 such patients provided by two physicians in private practice is summarized and presented in Table 2.

**Table 2:** Clinical histories of 14 patients of spirochetemia with low bacterial density.

Case #	Sex, Age, Disease Duration (in Years)	Major Clinical Presentations	Ser Test	Antibiotics 3–4 Weeks X	Response to Antibiotics	S/S 02/14	DNA Sequencing Showed in Blood 02/2014
1	M, 30 (2 years)	Rash, Neurol	−	1	Short term	Neurol	*B. miyamotoi*
2	M, 36 (3 years)	MusAc, Fatig	−	none	NA	MusAc	*B. burgdorferi*
3	M, 16 (3 years)	Flu	≠	1	Short term	Psy	*B. burgdorferi*
4	F, 24 (<1 year)	Flu	−	<1	Short term	Neurol	*B. burgdorferi*
5	F, 48 (15 years)	Flu	+	1	Short term	Neurol	*B. burgdorferi*
6	M, 18 (1 year)	Neurol	−	None	NA	Neurol	*B. burg. + B. miyam.* (Figure 8)
7	F, 22 (10 years)	Neurol, Fatig	−	>6	Short term	MusAc	*B. burgdorferi*
8	M, 11 (7 years)	MusAc	+	>1	Short term	MusAc	*B. burgdorferi*
9	F, 59 (20 years)	Rash, Fatig	+	None ^#^	NA	Fatig	*B. burgdorferi*
10	F, 49 (5 years)	Rash, Fatig	−	None	NA	Fatig	*B. miyamotoi*
11	F, 71 (2 years)	MusAc, Fatig	+	>2	Short term	Fatig	*B. burgdorferi*
12	F, 26 (20 years)	Flu, Neurol	ND	>5	Short term	Neurol	*B. burgdorferi*
13	M, 66 (2 years)	Flu, Neurol	ND	>3	Minimal *	Fatig	*B. burgdorferi*
14	M, 69 (13 years)	Neurol. Flu	−	>3	Short term	Fatig	*B. miyamotoi*

The above 14 patients visited two physicians, one in Massachusetts (cases #1–7 to K.L.), one in Connecticut (cases #8–14 to T.A.M.) for suspect Lyme disease in the month of February 2014 when there was little tick exposure in the region. All blood samples were drawn between 21 January and 3 March 2014. The information summarized in Table 2 was extracted from the patients’ past records. Major clinical presentations were the initial symptoms/signs including skin rash (possible Erythema migrans), flu-like symptoms (Flu), muscle aches or joint pain (MusAc), neurological problems (Neurol), psychiatric problems (Psy) and fatigue (Fatig). Ser Test = the 2-tier serology tests, including Western blots for IgG and IgM bands recommended by the CDC; +: positive; −: negative; ND: not diagnostic. Antibiotics, if given, were at least 3–4 weeks in one course. The kinds of antibiotics prescribed were too many to list here, and the number of courses of antibiotics was usually given for more than one course (>1). NA: not applicable. S/S 02/14 = major symptom/sign on the date when blood was drawn for DNA sequencing. ≠: Patient #3 had initially flu-like symptoms in summer 2010, then developed bilateral knee swelling in February 2011. After a serology test was interpreted as positive for Lyme disease, the patient was treated with doxycycline for 28 days and the arthritis resolved. About 12 months after that treatment, the patient started having multiple physical and psychiatric symptoms, and eventually in the summer 2013 saw a Lyme disease expert who interpreted the ELISA test to be positive, but the Western blots negative because the Western blots showed negative IgM and positive IgG for 6 bands. Based on the expert’s opinion, the patient was hospitalized for pure psychiatric illnesses at a psychiatric hospital for 7 weeks in the fall of 2013. The patient also had multiple other health issues, including typical Bartonella rash, sweats, flushing, severe fatigue, migratory joint pains, nausea, stomach pain, insomnia and blurry vision. He did not attend school for one year 2013–2014. He was discharged in the fall of 2013 and was not diagnosed as a Lyme disease patient until February 2014 when the patient also had a positive C6 peptide *B. Burgdorferi* IgG/IgM 3. 46 Lyme Index (normal range < 0.91), in addition to DNA sequencing positive for *B. burgdorferi.* #: Patient #9 could not tolerate antibiotic treatments. *: Patient #13 was on antibiotic treatment when the sample was drawn. He had minimal positive response while on antibiotic treatments. Symptoms exacerbated quickly off antibiotics.

As mentioned above, the bacteria isolated from the blood samples of these 14 patents were pelleted by differential centrifugation and concentrated. The extracted bacterial chromosomal DNA was validated by analysis of at least 100 bases of a 16S rDNA signature sequence immediately downstream of the M1 primer-binding site through BLAST alignment algorithms. A 100% ID match between the sample sequence and a standard *B. burgdorferi* or *B. miyamotoi* DNA sequence stored in the GenBank databases was required for establishing the diagnosis. The sequencing data have provided molecular evidence beyond reasonable doubt that some bacteria of *B. burgdorferi* and *B. miyamotoi*, dead or alive, did exist in the circulating blood of these ambulatory patients living in the northeastern states of the U.S.A. in a deep cold winter month. Since new infections of Lyme and related borrelioses are primarily seasonal and unlikely to occur in such small clusters in one New England deep winter month when tick activity is highly limited, these off-season spirochetemias with low bacterial densities were most likely the result of the bacteria being dislodged periodically from persistent deep tissue lesions [32,33,34].

Full-blown spirochetemia in Lyme borreliosis is a transient phenomenon and occurs within the first 30 days of the disease [35]. Any free bacterial DNA in the circulating blood left over from an early full-blown spirochetemia would be degraded or excreted within 42 h [36] after the spirochetes vanished from the circulating blood, and certainly would have been excluded when the bacteria were pelleted by differential centrifugation to be tested according to our laboratory procedures. Since the spirochetemia detected in these 14 patients was unlikely to be the result of a recent infection and some of the patients had received multiple courses of antibiotics for the treatment of the disease up to the date of blood testing, we interpret these 14 patients as cases of undiagnosed Lyme or related borrelioses, or as cases of “Lyme disease” not completely cured by the standard courses of antibiotic treatment.

The high proportion of *B. miyamotoi* infections (4/14) among the patients listed in Table 2 was a surprise because only three hospitalized patients of *B. miyamotoi* infection have been reported in the U.S. [15,16]. Infection prevalences in questing nymphal ticks are known to range between 0.20 and 0.50 for *B. burgdorferi* and 0.01 to 0.05 for *B. miyamotoi* by PCR [37,38]. However, a recent serologic study reported that the ratio of *B. miyamotoi*: *B. burgdorferi* human infections in southern New England may be as high as 25:60 [39], suggesting that there may be many undiagnosed *B. miyamotoi* infections in this region. The true number of ambulatory patients infected with *B. miyamotoi* remains unknown until a sensitive, reliable diagnostic laboratory test is available to detect the causative agents in the patients with acute infections in the endemic areas.

### 2.5. Detection of Co-Infection by B. miyamotoi and B. burgdorferi in Patient with Spirochetemia

**Figure 8:**
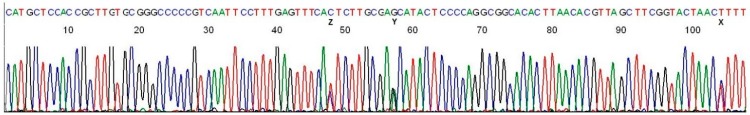
A 107-base segment of the base-calling DNA sequencing electropherogram showing two superimposed DNA sequences, one of *B. burgdorferi* and one of *B.* *miyamotoi*. There are three characteristic double base peaks at positions 770, 815 and 826 (Table 1) in this signature sequence, representing concomitant borrelial infections by these two species in one patient blood sample. The M2 primer was used as the sequencing primer.

One patient (case #6, Table 2) was found to be co-infected with a *B. burgdorferi* and a *B. miyamotoi* by analysis of a 107-base sequence downstream of the M1 primer site (Figure 8).

This electropherogram illustrated two highly conserved sequences, one on top of the other, with three ambiguous bases at positions marked by the letters X, Y and Z below the computer-generated nucleotide bases downstream of the M1 primer-binding site (reading from right end to left. The M1 primer has been cut off). A visual analysis revealed that the ambiguous base in position X in fact consists of a base “C” and a base “T”, the ambiguous base in position Y a base “A” and a base “G”, and the ambiguous base in position Z a base “T” and a base “C”. This can be recognized more clearly by alignment of the sequence in Figure 8 against those in Figure 2 and Figure 3. By permutations of the three double bases at positions Z, Y and X (yellow-highlighted), the mixed sequences in Figure 8 may be the result of any pairing of eight possible individual DNA sequences listed as follows.


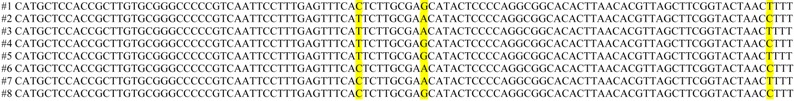


Submission of each of these eight sequences to the GenBank for BLAST alignment analysis confirmed that only sequence #1 and sequence #2 have a 100% ID match with a natural 16S rDNA segment of *B. burgdorferi* and *B. miyamotoi*, respectively. Sequences #3 through #7 do not have a 100% ID match with any sequences stored in the GenBank. The presence of a base “T” in position X of this 107-base sequence #1 establishes a sequence of *B. burgdorferi* in the sample. Sequence #8, if present, could have been generated by the 16S rDNA from some strains of the relapsing fever Borrelia group listed in Table 1 except *B. miyamotoi* which has its exclusive unique invariant bases in positions 817 and 826 (Table 1)*.* None of the known relapsing fever borreliae other than *B. miyamotoi* can generate a DNA sequence with two bases of “A” and “G” in position Y in combination with a strain of *B. burgdorferi* sensu lato as shown by this mixed DNA sequence. By excluding all impossibilities, we concluded that the nested PCR products illustrated in Figure 8 were composed of two superimposed borrelial 16S rDNA sequences, one from *B. miyamotoi* and one from *B. burgdorferi* beyond a reasonable doubt.

Since the M1/M2 amplicon of *B. burgdorferi* is one base shorter than that of *B. miyamotoi* because of a base gap near the end of its M1 primer-binding site (Table 1), the molecular ladders generated by Sanger reaction on the two sequencing templates cannot be deciphered by the computer when the M1 primer is used as the sequencing primer due to reading frame shifting.

Patient #6 is the first known human case co-infected by a *B. burgdorferi* and a *B. miyamotoi* which is proved by DNA sequencing analysis although five (0.009) of 556 mouse blood samples were found to be infected with both of these two Borrelia species by qPCR in a Connecticut field study [17]. More human co-infections may be expected if testing methods for detecting both Borrelia species are implemented widely.

## 3. Experimental Section

### 3.1. Sources of Borrelial DNA

The cells of *B. burgdorferi* sensu stricto, strain B31 (ATCC 53210) in liquid culture purchased from American Type Culture Collection, Manassas, VA, USA, were pelleted and the DNA was extracted by ammonium hydroxide solution. The crude DNA was precipitated by ethanol and used as the standard control according to the procedure previously described [13]. Numerous archived *Ixodes scapularis* ticks which were removed by the local patients from the skin at the site of the tick bite were extracted by hot ammonium hydroxide and the crude DNA in solution was precipitated by ethanol, and screened by various PCR primers known to be capable of amplifying a segment of 16S rDNA of the species of *B. miyamotoi.* The crude extracts from the ticks containing species-specific 16S rDNA of *B. miyamotoi* confirmed by Sanger DNA sequencing were used as the positive control for development of the diagnostic PCR for *B. miyamotoi*.

### 3.2. Blood Sample Preparation

It was previously reported that for molecular diagnosis of spirochetemia the nested PCR amplification technology may raise the sensitivity of the conventional PCR detection of *B. burgdorferi* by 100–1000 fold and is capable of detecting a single copy of borrelial 16S ribosomal RNA gene after a primary PCR followed by a nested PCR in tandem [13]. However, most of the spirochetes in the whole blood samples may be trapped in the blood clot during serum preparation or lost from the plasma routinely prepared for chemistry analyses by high-speed centrifugation. As a result, unspun plasma separated from the red cells by gravitation sedimentation was chosen as the sample for PCR amplification if spirochetemia was suspected. The limit of detection was estimated to be about 1000 bacterial cells per mL of blood. In practice, as the copy number of the target bacterial DNA in the PCR mixture decreases to approaching the threshold level of detection, the PCR-inhibitory effects of the human genomic DNA derived from the white blood cells became pronounced, probably through non-specific competitive primer-binding [26,27]. Therefore, we decided to use differential centrifugation to separate the spirochetes from the blood cells, and then the spirochetes were spun down by high speed centrifugation along with the platelets in order to exclude most of the human genomic DNA in the whole blood samples.

For procedural development, we first centrifuged samples of normal human whole blood with EDTA anticoagulant in a low-speed Eppendorf micro centrifuge model # 5702 equipped with an A-4-38 rotor and four adapters for test tubes with 13 mm outer diameter at various *g* forces and times to obtain the platelet-rich plasma. Preliminary work on whole blood samples spiked with pure culture of *B. burgdorferi* confirmed that the density gradient of *B. burgdorferi* is closer to that of the platelets. The spirochetes floating in the platelet-rich plasma can be spun down in a high-speed Eppendorf micro centrifuge model # 5424 equipped with a 24 × 1.5/2 mL fixed angle rotor. By testing the serial dilutions of the borrelial DNA extracted from the pellet of the platelet-rich plasma derived from whole blood samples spiked with a fixed number of *B. burgdorferi* pure culture cells, it was found that more than 90% of the borrelial cells used to spike the whole blood samples were consistently recovered from the platelet pellet. The protocol for routine differential centrifugation for spirochete concentration described below was based on the results of these experiments.

In practice, venous blood collected from patients with suspect Lyme disease in a lavender top test tube containing EDTA anticoagulant was shipped to the laboratory at ambient temperature via overnight courier delivery. The blood samples received were stored in a 4 °C refrigerator and usually processed within 48 h. On the day of testing, the blood sample was warmed up to room temperature, mixed well and first centrifuged at ~400× *g* (1400 rpm) for 15 min to spin down the red and white cells. One mL of the platelet-rich plasma was transferred to a 1.5 mL plastic tube to be further centrifuged at ~16,000× *g* (13,000 rpm) for 10 min. to collect the platelets and the spirochetes, if any, in the pellet. After the supernatant was discarded, the pellet was suspended in 100 µL of tris(hydroxymethyl)-aminomethane hydrochloride–EDTA (TE) buffer, pH 7.5, and 200 μL 0.7 M ammonium hydroxide. The mixture was heated at 95–98 °C for 5 min. with closed cap, followed by 10 min. with open cap. After the test tube was cooled to room temperature, 30 μL of 3 M sodium acetate and 700 μL of ice-cold 95% ethanol were added to the mixture. The mixture was centrifuged at 13,000 rpm (~16,000× *g*) for 5 min. and the supernatant discarded. The precipitate was re-suspended in 1 mL of cold 70% ethanol. Then the suspension was centrifuged at 13,000 rpm for 5 min. After all liquid was discarded, the pellet was air-dried, re-suspended in 100 µL TE buffer and heated at 95–98 °C for 5 min. The heated suspension was finally centrifuged at 13,000 rpm for 5 min. One µL of the supernatant without further purification was used to initiate each primary PCR to be followed by nested PCR amplification. To test for borreliae in patients of spirochetemia with low bacterial density, we routinely perform four primary PCRs followed by four nested PCRs for each blood sample, plus one negative deionized water control and one positive *B. burgdorferi* control on each set of primary PCRs and nested PCRs.

Since the spirochetes in 1 mL of platelet-rich plasma were all pelleted for DNA extraction and the volume of final DNA extract was reduced to 100 µL, the borrelial 16S rDNA in the final DNA extract was 10-fold concentrated as compared to using plasma without prior concentration for DNA extraction. If the final DNA extract contained exactly one copy of borrelial 16S rDNA (limit of detection) in every 1 µL aliquot, the original plasma sample must contain 100 spirochetes per mL. If only one of the four 1 µL aliquots pipetted out from a final DNA extract to initiate each of the four primary PCRs turned out to be positive for 16S rDNA in the nested PCR (see below), the collective nested PCR results would indicate that the original plasma sample contained ~25 spirochetes per mL, which is the *in vitro* sensitivity of the test. We consider this amplification to be highly sensitive.

### 3.3. PCR and DNA Sequencing Primers

Initially, numerous species-specific oligonucleotides were synthesized as PCR primers in order to find a tick extract positive for *B. miyamotoi* chromosomal DNA to be used as the template for methodology development. Five nucleotides were used as diagnostic PCR and DNA sequencing primers, including the species-specific LD1 (5'-ATGCACACTTGGTGTTAACTA-3') and LD2 (5'-GACTTATCACCGGCAGTCTTA-3') PCR primer pair [10,11], the TEC1 (5'-CTGGGGAGTATGCTCGCAAGA-3') heminested PCR primer [12], and the newly designed M1 (5'-ACGATGCACACTTGGTGTTAA-3') and M2 (5'-TCCGACTTATCACCGGCAGTC-3') general primer pair.

The M1/M2 primer pair was designed to generate a 357-bp PCR amplicon from all species in the *B. burgdorferi* sensu lato complex and a 358-bp PCR amplicon from *B. miyamotoi*. In a previous report, the M1/M2 primer pair has been shown to be able to amplify a correspondent 358-bp segment of the 16S rDNA of *B. coriaceae* and a correspondent 358-bp segment of the 16S rDNA of a novel Borrelia, both belonging to the relapsing fever Borrelia group, as the species of *B. miyamotoi* [25].

### 3.4. PCR Conditions

For the positive controls, 1 µL of the crude DNA extract of the standard *B. burgdorferi* culture containing 10 copies of borrelial chromosomal DNA in TE buffer was used to start the primary PCR to be followed by heminested PCR or same-nested PCR amplification with a pair of proper primers, using a ready-to-use LoTemp^®^ PCR mix catalyzed by a moderately heat-resistant DNA polymerase [40] in a total volume of 25 µL per PCR. Briefly, for amplification of the highly conserved segment of the borrelial 16S rDNA, 1 μL of the DNA extract was added into a PCR tube containing 20 μL of ready-to-use LoTemp^®^ PCR mix (HiFi DNA Tech, LLC, Trumbull, CT, USA), 2 µL of deionized water, 1 μL of 10 μmolar forward primer, and 1 μL of 10 μmolar reverse primer to initiate a primary PCR. The thermocycling steps were programmed to 30 cycles at 85 °C for 30 s, 50 °C for 30 s, and 65 °C for 1 min after an initial heating for 10 min at 85 °C, with a final extension at 65 °C for 10 min. A trace of each of the primary PCR products was transferred by a micro-glass rod to another 25 μL complete PCR mixture containing 20 μL of ready-to-use LoTemp^®^ PCR mix, 1 μL of 10 μmolar forward primer, and 1 μL of 10 μmolar reverse primer and 3 μL of water for heminested PCR or same-nested PCR amplification with identical thermocycling steps as for the primary PCR. To perform heminested PCR, the primers used were a TEC1/LD2 primer pair, or a TEC1/M2 primer pair. For same-nested PCR, the primary PCR and the subsequent nested PCR(s) were conducted with an identical pair of M1 and M2 PCR primers so that all PCR amplicons were terminated by the sequences of the PCR primers used to initiate the primary PCR [25].

To test for borreliae in the patient samples and in the archived ticks, 1 µL of crude DNA extract of the platelet-rich plasma pellet or of a tick was used, instead of the standard positive control DNA extract, to initiate the primary PCR.

### 3.5. DNA Sequencing

The nested PCR amplicon was transferred by a micro-glass rod into a Sanger reaction tube containing 1 μL of 10 μmolar sequencing primer, 1 μL of the BigDye^®^ Terminator (v 1.1/Sequencing Standard Kit), 3.5 μL 5× buffer, and 14.5 μL water in a total volume of 20 μL for 20 enzymatic primer extension/termination reaction cycles according to the protocol supplied by the manufacturer (Applied Biosystems, Foster City, CA, USA). After a dye-terminator cleanup with a Centri-Sep column (Princeton Separations, Adelphia, NJ, USA), the reaction mixture was loaded in an automated ABI 3130 four-capillary Genetic Analyzer for sequence analysis. Sequence alignments were performed against the standard sequences stored in the GenBank databases by on-line BLAST alignment analysis [41]. The M2 primer was routinely used for DNA sequencing to obtain a >100 base unambiguous sequence immediately downstream of the M1 primer. A 100% ID match between the sample sequence and the standard sequence in the GenBank [41] was required for molecular diagnosis of a *B. burgdorferi* sensu lato or a *B. miyamotoi.*

The usefulness of 16S rDNA sequencing as a tool in microbial identification is dependent upon two key elements, deposition of complete unambiguous nucleotide sequences into public or private databases and applying the correct “label” to each sequence [42]. Repeated re-amplifications of a 16S rDNA PCR amplicon are known to be associated with laboratory-induced mutations in the final PCR amplicons [43,44,45], and may cause difficulties in data analysis if the PCR amplicons are used as the template for direct DNA sequencing. We have chosen a moderately heat-resistant high-fidelity DNA polymerase to perform PCR at low thermocycling temperatures not to exceed 85 °C [40] to reduce the rates of PCR-induced sequence artifacts and bias which may occur in same-nested PCR amplifications.

### 3.6. Cross Contamination Control

Cross-contamination is a serious concern in any clinical laboratories performing nested PCR DNA amplification. However, cross-contamination is not an inherent part of the nested PCR technology. It is rather a function of the clinical laboratory that performs PCR.

To reduce the chances of cross contamination, three separate rooms with no air re-circulation were dedicated to the nucleic acid amplification tests. Two of the rooms were each equipped with a 32" PCR workstation (AirClean Systems, Raleigh, NC, USA). All pre-amplification procedures were performed in PCR station I. All post-PCR procedures were carried out in PCR station II, including preparations for the nested PCR and sequencing reaction. Gel electrophoresis and DNA sequencing were performed in the third isolation room. No post-PCR materials or any items potentially contaminated by PCR amplicons or equipment used in the post-PCR rooms were allowed to enter the pre-PCR working space.

Transferring of PCR products from one test tube to another was always accomplished by a standardized micro-glass rod to avoid PCR product aerosol induced by micropipetting [46]. Before being allowed to work independently after a course of hands-on practical training, all technologists passed an in-house proficiency test. The latter consists of performing nested PCR on a single batch of 50 simulated samples, about one third (1/3) of which have been randomly spiked with a target DNA, and requires a 100% correct result to pass.

All the testing procedures were performed physically in the Molecular Diagnostics Department of Milford Medical Laboratory, Inc., an affiliation of Milford Hospital, licensed to perform these high-complexity diagnostic tests by the Connecticut State Department of Public Health under the Clinical Laboratory Improvement Amendments of 1988 regulations (CLIA). The final results, if positive for spirochetemia, are routinely reported with an accompanying DNA sequencing electropherogram and GenBank BLAST alignment analysis between 48 and 72 h from the start of sample processing. Negative results are reported within 48 h.

## 4. Conclusions

By differential centrifugation, the sparse bacterial cells of the infectious agents in the blood of the patients with Lyme and related borrelioses can be pelleted for nucleic acid amplification test. Using a pair of general primers for same-nested PCR amplification of a target 16S rDNA sequence of *B. burgdorferi* and *B. miyamotoi* for detection and to be used for Sanger DNA sequencing, the presence of these spirochetes in the pellet of the platelet-rich plasma can be confirmed beyond reasonable doubt. With this newly developed method, we found 14 ambulatory patients with spirochetemia containing 25–50 borrelial cells per mL of whole blood in a month of deep winter in the Northeast of the U.S.A. when tick activity in the region was minimal. We conclude that these patients represented undiagnosed cases of “Lyme and related borreliosis” or persistent infection of “Lyme and related borreliosis” after standard antibiotic treatments failed to eradicate the causative agents from the infected tissues. A sensitive and reliable laboratory test for the infectious agents of “Lyme and related borreliosis” should be available in the hospital laboratories located in the disease-endemic communities to diagnose these cases for timely and appropriate treatment.
